# Distinct mechanism of formation of the 48, XXYY karyotype

**DOI:** 10.1186/1755-8166-6-25

**Published:** 2013-07-03

**Authors:** Aránzazu Margallo Balsera, Manuela Núñez Estévez, Emilia Balboa Beltrán, Plácida Sánchez-Giralt, Luz González García, Trinidad Herrera Moreno, Mayte García de Cáceres, José M Carbonell Pérez, Enrique Galán Gómez, Raquel Rodríguez-López

**Affiliations:** 1 Genetics Unit, Infanta Cristina Hospital, Carretera de Portugal S/N, Badajoz 06080, Spain; 2 Unit of Genetic Pediatrics, MaternoInfantil Hospital de Badajoz, Badajoz 06010, Spain; 3 Department of Dietician, Endocrinologist Service, Infanta Cristina Hospital, Badajoz 06080, Spain

**Keywords:** 48,XXYY, 47,XYY, Mechanism origin, Paternal, Spermatogenesis

## Abstract

**Background:**

To expose the unusual nature of a coincident sex chromosomal aneuploidy in a patient and his father. Molecular mechanisms involved probably are based on the sperm chromosome of paternal origin, which determine the mode of formation. Conventional cytogenetics techniques and multiple Quantitative Fluorescent PCR of STR markers in sexual chromosomes in the patient and his parents.

**Results:**

48,XXYY and 47,XYY aneuploidies in the patient and his father, respectively, were identified. The additional X and Y chromosomes showed parental origin.

**Conclusions:**

An infrequent origin of the 48,XXYY syndrome was demonstrated. Mostly, it is thought to result from an aneuploid sperm produced through two consecutive non disjunction events in both meiosis I and II in a chromosomally normal father, but in our father’s patient a 47,XYY was discovered. It is suggested that a higher incidence of 24,XY and 24,YY sperm may be possible in 47,XYY individuals andan increased risk for aneuploidy pregnancies may exist. Although 48,XXYY patients and Klinefelter syndrome are often compared, recently they are regarded as a distinct genetic and clinical entity.

## Background

The 48,XXYY Syndrome is an uncommon sex chromosome aneuploidy condition that is characterized by the presence of one extra X and Y chromosomes in males. Until recently, XXYY Syndrome was considered as a variant of the Klinefelter syndrome (which includes the karyotypes 47,XXY; 48,XXYY; 48,XXXY and 49 XXXXY), but numerous authors have become aware that it has distinctive features. 48,XXYY syndrome the first observation was in 1960 by Muldal and Ockey [1] and later by Court Brown et al. [2] was described in 1964 and occurs in an estimated 1:18,000–1:50,000 males [3]. The patients show similar features of both individuals carrying 47,XXY and 47,XYY karyotypes [4], including tall stature, narrow shoulders, broad hips, sparse body hair, gynecomastia, normal to slightly decreased verbal intelligence [4,5] and hypergonadotropichypogonadism and infertility based on: small testes, absentspermatogenesis, normal to moderately reduced Leydig cell function, increased secretion of follicle-stimulating hormone and androgen deficiency [4-6].

When 48,XXYY and 47,XXY patients are clinically compared some differences are found: The first condition shows more frequently facial dysmorphism and congenital malformations [7] and also have a lower IQ with frequent and severe behavioral and psychiatric problems, including attention deficit hyperactivity disorder, autism spectrum disorders, and mood and tic disorders [6,7]. Males with sex chromosomal aneuploidies are known to have variability in their developmental profile with the majority presenting with expressive language deficits [6-8].

During the formation of the sperm, aneuploidy of the sex chromosomes is more frequent than aneuploidy of any of the autosomes and an association has been established between increased sperm aneuploidy and male subfertility [9].

A direct relationship between the number of additional sex chromosomes and the severity of the phenotype is generally assumed [10].

Some studies suggest that the frequency of XY sperm increases with age in parents of children with Klinefelter syndrome [11]. If one of these atypical sperm contributes to the genetic makeup of the child, it will have an extra X and Y chromosome in every cell.

47,XYY patients have a prevalence of approximately 0.1% of male population with the extra Y chromosome resulting from paternal non-disjunction at meiosis II. Usually they are fertile, with most of sperm cells with a normal karyotype. It has been hypothesized that one of the two Y chromosomes is lost before entering spermatogonia in meiosis. Several sex chromosome pairing configuration have been described at the pachytene stage: X + YY (most frequent), XY + Y, X + Y + Y and XYYtrivalent [12-14]. Only XYY trivalent configuration is believed to produce sex chromosome aneuploid sperm. An inverse correlation has been found between the proportion of XYY viable germ cells and low sperm counts with elevated sperm sex aneuploidies. Sex chromosome aneuploidies are more frequently of paternal origin as follow: 50% of 47,XXY, 100% of 47,XYY and 70-80% of 45,X [15].

Most of 48,XXYY are thought to result from an aneuploid sperm produced through two consecutive non disjunction events in both meiosis I and II in a chromosomally normal father, so a boy with XXYY has one X chromosome from his mother and the additional XYY from his father.

We reported a case of a child with karyotype 48,XXYY, whose neurodevelopment delay, poor motor coordination, behavioral problem, facial features and unilateral kidney was the indication for genetic testing at 7 years of age. One case of 48,XXYY syndrome with unilateral renal aplasia has been previously reported [16].

Father’s karyotype was determined and surprisingly, the result was 47,XYY. This is the first case of a 48,XXYY patient whose father carries a sex aneuploidy. Parental origin and hypothesis about the origen of the additional X and Y chromosomes in the patient were performed based on molecular data.

## Results

**Figure 1:**
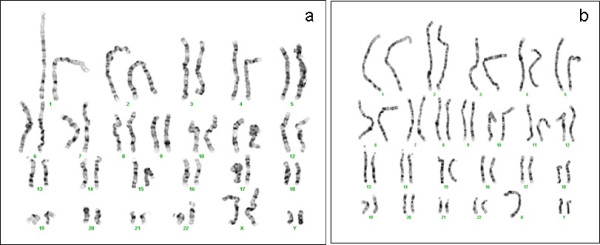
**Image of the karyotypes of the patient (a) and his father (b).** The patient’s karyotype showed the existence of extra Chromosomes X and Y, with a formula of 48, XXYY. The father’s karyotype turned out to be a carrier of the disorder XYY.

The QF-PCR results confirmed the obtained data extracted by using the conventional cytogenetics techniques. The abnormal karyotypes with additional X and/or Y chromosomes: the 48,XXYY and 47,XYY karyotypes were found in all metaphases of the patient and his father, respectively (Figure 1).

**Figure 2:**
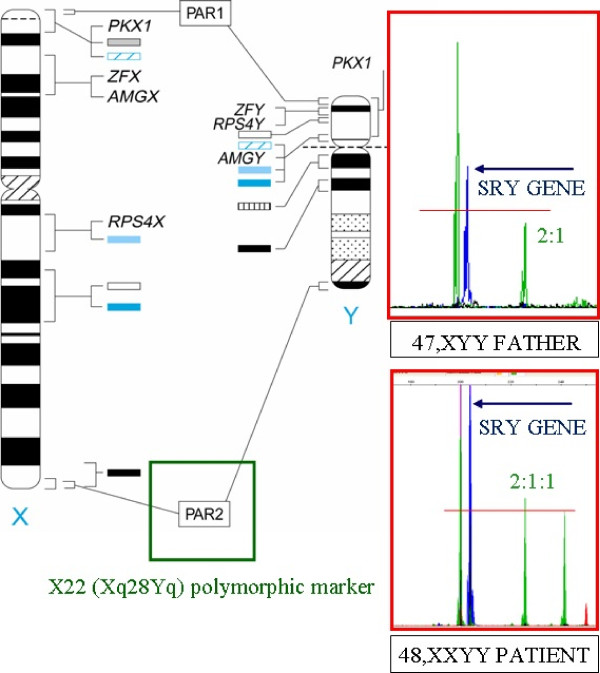
**Analysis aneuploidies of chromosomes X/Y by QF-PCR.** Analysis of polymorphic markers through Chromoquant Kit in chromosomes X and Y, as well as genes AMEL X/Y and SRY chain reaction quantitative fluorescent polymerase (QF-PCR) showed an envelope in 2X/2Y 1X/2Y the patient and his father. The figure shows SRY gene amplification and the marker image X22 (2:1:1 and 2:1 in the patient and his father).

The mother had a normal karyotype 46,XX and also the patient’s brother showed anormal 46,XY karyotype. Size in base pairs and ratios for the area corresponding to the peaks of the fluorescence signal detected X-chromosome markers amplified specific STR, demonstrated that the patient had received one X chromosome from the mother and the other from the father. The extension of the molecular characterization (Table 1) of the 48,XXYY karyotype to his parents revealed the origin is paternal, as is generally accepted in this syndrome. The Figure 2 shows the marker image X22 (2:1:1 patient) and (2:1 patient’s father) and SRY gene amplification. In the (QF-PCR) showed an envelope in 2X/2Y patient and 1X/2Y his father. This results in a sperm with three sex chromosomes (one X chromosome and two Y chromosomes).

**Table 1:** Molecular and cytogenetic characterization

	**Patient**	**Father**	**Mother**
KARYOTYPE	48,XXYY	XYY (QF-PCR)	XX (QF-PCR)
AMEL (Xp22.31-Xp22.1/Yp11.2)	XY	XY	X
SRY (Yp11.31)	Present	Present	No
X22 (Xq28Yq)	199/225/241 bp(2:1:1)	199/225 bp(2:1)	220/241 bp
DXS6854 (Xq26.1)	110/110 bp	110 bp	106/110 bp
XHPRT (X26.1)	274/285 bp	285 bp	274/281 bp
DXS6803 (Xq21.31)	110/120 bp	110 bp	120/120 bp
DXS8377 (Xq28)	250/262 bp	250 bp	253/262 bp
DXS6809 (Xq21.33)	263/271 bp	263 bp	267/271 bp
G10_STS47 (Yq11.222)	Present	Present	No

Set of DNA fragment containing genes and genetic markers Short Tandem Repeat (STR) analysed, using Quantitative Fluoresce PCR in the patient and his parents. Parental alleles of the polymorphic markers evidenced that the additional X chromosome of the patient came from the father.

We propose two possible origins of the extra sex chromosomes in the patient (spermatogonia with a normal karyotype or with an extra Y chromosome).

## Discussion and conclusions

Molecular and cytogenetic characterization of the patient carrying the 48,XXYY karyotype indicated a paternal origin of the additional X and Y chromosomes. Although there appears to be no particularly increased risk that a 47,XYY male will have a chromosomally abnormal child, it is not possible to discard the effect of the paternal aneuploidy in this patient.

It is accepted that XYY spermatogonia are able to produce altered 24,XY and 24,YY spermatozoa, although the ejaculate of XYY men have generally shown a very small increased fraction of them. In a small percentage of patients, mitotic non-disjunction occurring during the early stages of a 46, XY zygote can result in 48,XXYY syndrome [17].

We propose two possible origins of the extra chromosomesin 48,XXYY patient: it is accepted that extra chromosome Y is lost in most of primary spermatocytes (pachytene cells) of 47,XYY patients. Also, it has been found that a high proportion of XYY configuration at pachytene in testis is negatively correlated with the sperm count in ejaculates [18,19], so the minority of oligospermic patients has a higher incidence of disomic 24,XY and 24,YY spermatocytes.

In our patient’s father there is no history of infertility so we can think that the extra Y chromosome is lost in most of spermatocytes. In those chromosomally normal sperm precursors (46,XY cells), two consecutive nondisjunction events in both meiosis I and II could originate an abnormal 25,XYY sperm. This option also discards the implication of the karyotype character of the father in the appearance of sex chromosome abnormality in the child.

As we have commented previously, several sex chromosome pairing configurations have been described at the pachytene stage, and only XYY trivalent configuration is believed to produce sex chromosome aneuploidy. Spermatocytes with a trivalent configuration are compatible with the progress of meiosis [20,21]. Other sex chromosome configurations are not observed in Spermatocytes I with apoptosis occurring at the testicular level [19]. In the case of XYY configuration, we can speculate that a single non disjunction event of all gonosomes in meiosis I of these cells could also originate a 25,XYY sperm. If this has been the mechanism involved in our patient, an increased risk for aneuploidy pregnancies may exist.

The QF-PCR technique resulted extremely sensitive to detect X and Y chromosome anomalies in postnatal diagnosis. The case presented by Zhang and Li (2009) [22] also shows that QF-PCR assays can provide rapid prenatal diagnosis of numerical sex chromosome aneuploidy.

It has been suggested that additional molecular data gained increased importance in this variant of Klinefelter syndrome, in which distinct patterns of X inactivation could play a role in the observed differences in the degree of clinical manifestations of patients. The wide range of psychological and medical problems has been attributed to skewed X chromosome inactivation [23].

Metabolic syndrome is associated with insulin resistance, the leading cause of truncal obesity and muscle wasting, which was observed in the described patient. Also considered an endocrine disorder [24,25] and the risk of developing type 2 diabetes is high.

48,XXYY resembles Klinefelter’s syndrome although they are now considered differententities. It has been suggested that parent of origin of extra chromosome in 47,XXY influences the phenotype. Additional studies in 48,XXYY patients are needed to elucidate any epigenetic factors affecting the phenotype. Increased awareness of the developmental, psychological, and medical features of 47,XXY and 48,XXYY is important to facilitate timely diagnosis and initiation of appropriate screenings and treatments that are important for optimal outcomes [4,8,26].

## Material and methods

### Patient

The patient was a Caucasian Spanish 7 year old, who was referred to the Genetics Unit of the Infanta Cristina Hospital in Badajoz. This patient was referred for karyotyping due to hypergonadotropichypogonadism, tall stature, and other associated features include neurodevelopment delay, poor motor coordination, behavioral problem, facial features and unilateral kidney, specifically, anorchia with unilateral renal agenesis. Right orchidopexy intervene exeresis of the left cord.

The patient has a long face, epicanthal folds, hypertelorism, narrow palpebral fissures, full lips and dental defects. Overweight was evident at 5 years of age and obesity from the 7 (3.6 standard deviation in weight), parents are not obese. The psychomotor and neurocognitive developmental were normal. No deviations were observed in progression in the areas of communication and socialization, language and motor adaptive wide. For all this clinically suspected to have Klinefelter syndrome.

### Methods

After obtaining informed consent of the parents’ proband, peripheral blood was extracted and processed by standard techniques for conventional cytogenetic and molecular analysis. Quantitative fluorescent PCR (QF-PCR) assay were carried out in the patient, her parents and his unique brother. Chromosomal analysis was performed using standard techniques. Giemsa-based chromosomal banding and staining techniques detected the numerical chromosomal abnormalities.

Genomic DNA was obtained from the patient, parents and brother. Multiple quantitative fluorescent PCR (QF-PCR) was used including the amplification of amelogenin (AMELX and AMELY), which is presented on both sex chromosomes in a biallelic form, SRY gene (sex-determining region Y), a polymorphic short tandem repeat (STR) on the pseudoautosomal region of X and Y (X22), five polymorphic X-specific STRs (DXS6803, XHPRT, DXS6803, DXS8377 and DXS6809), and a Y-specific marker (G10_STS47). STRs result in fragment length and area ratio differences which were analyzed with Applied Biosystems 3130 Genetic Analyzer *and GeneMapper*® Software *Version 4*.*0*. GeneMapper performed the identification of DNA fragments of interest and calculated their size in nucleotides, height and peak area. The size alleles corresponding to these peaks were identified and the results obtained in the child were compared and attributed to paternal or maternal ones for each marker; the ratio of heights or peak areas concerned.

## Competing interests

The authors declare that they have no competing interests.

## Authors’ contributions

RRL and JM.CP conceived the study and participated in its design and coordination. Helped to draft the manuscript. AMB carried out the molecular genetic studies, participated and drafted the manuscript. MGC, THM and EBB participated in the study design and protocol development. All authors read and approved the final manuscript.
